# Why Are There Social Gradients in Preventative Health Behavior? A Perspective from Behavioral Ecology

**DOI:** 10.1371/journal.pone.0013371

**Published:** 2010-10-13

**Authors:** Daniel Nettle

**Affiliations:** Centre for Behaviour and Evolution, Institute of Neuroscience, Newcastle University, Newcastle, United Kingdom; Swiss Paraplegic Research, Switzerland

## Abstract

**Background:**

Within affluent populations, there are marked socioeconomic gradients in health behavior, with people of lower socioeconomic position smoking more, exercising less, having poorer diets, complying less well with therapy, using medical services less, ignoring health and safety advice more, and being less health-conscious overall, than their more affluent peers. Whilst the proximate mechanisms underlying these behavioral differences have been investigated, the ultimate causes have not.

**Methodology/Principal Findings:**

This paper presents a theoretical model of why socioeconomic gradients in health behavior might be found. I conjecture that lower socioeconomic position is associated with greater exposure to extrinsic mortality risks (that is, risks that cannot be mitigated through behavior), and that health behavior competes for people's time and energy against other activities which contribute to their fitness. Under these two assumptions, the model shows that the optimal amount of health behavior to perform is indeed less for people of lower socioeconomic position.

**Conclusions/Significance:**

The model predicts an exacerbatory dynamic of poverty, whereby the greater exposure of poor people to unavoidable harms engenders a disinvestment in health behavior, resulting in a final inequality in health outcomes which is greater than the initial inequality in material conditions. I discuss the assumptions of the model, and its implications for strategies for the reduction of health inequalities.

## Introduction

Within economically developed countries, there are large differentials in long-term health outcomes between people of different socioeconomic positions [1], [2], [3]. The magnitude of these differences does not appear to be reducing over time [4], [5], [6], making them an important priority for public policy. Studies have consistently shown that preventative health behavior is a contributory factor to the inequalities in outcomes. People of lower socioeconomic position have been found to smoke more, exercise less, have poorer diets, comply less well with therapy, use medical services less, adopt fewer safety measures, ignore health advice more, and be less health-conscious overall, than their more affluent peers [7], [8], [9], [10], [11], [12], [13], [14], [15], [16], [17], [18]. Some of these behaviors can simply be put down to financial constraints, as healthy diets, for example, cost more than unhealthy ones, but socioeconomic gradients are found even where the health behaviors in question would cost nothing, ruling out income differences as the explanation. It is these health behaviors, which cost only time and energy, which are the focus of this paper. Socioeconomic gradients in health behavior are not easily abolished by providing more information. Informational health campaigns tend to lead to greater voluntary behavior change in people of higher socio-economic position, and thus can actually increase socioeconomic inequalities in health, even whilst improving health overall [19], [20]. Thus, we are struck with what we might call the exacerbatory dynamic of poverty: the people in society who face the greatest structural adversity, far from mitigating this by their lifestyles, behave in such ways as to make it worse, even when they are provided with the opportunity to do otherwise.

Underlying socioeconomic differences in health behavior are differences in attitudinal and psychological variables. People of lower socioeconomic position have been found to be more pessimistic [21], have stronger beliefs in the influence of chance on health [18], and give a greater weighting to present over future outcomes [22], [23], [24], than people of higher socioeconomic position. These explanations seem clear. However, they immediately raise the deeper question: why should pessimism, belief in chance, and short time perspective be found more in people of low socioeconomic position than those of high socioeconomic position? These deeper questions are at the level which behavioral ecologists call ultimate, as opposed to proximate causation [25]. That is, they ask why this suite of behaviors appears specifically in the environment of socioeconomic deprivation, rather than amongst the affluent. The general approach of behavioral ecology is to set up models of how individuals ought to be expected to behave, given the environments they experience, if they are in fact making optimal decisions. That is, we need to consider the possibility that the lower investment in health behavior in people facing socioeconomic deprivation is in fact adaptive given their circumstances.

Previous commentators have pointed out that there may be a rational basis to the neglect of preventative health behavior often seen in people of lower socioeconomic position. For example, the incentive to forego smoking is small for population sub-groups who are likely to die from some other cause anyway before the effects of their smoking lead to disease [26]. If we extend this argument to all health behaviors, though, there seems to be a danger of circularity, since we end up explaining neglect of health behavior by low life expectancy, whilst low life expectancy is itself caused, to a considerable extent, by neglect of health behavior. Thus, which is cause and which effect in the web of relationships needs to be clarified. Indeed, though the four-way correlation of socioeconomic position, attitudes, health behaviors and health outcomes is well documented, it remains true that epidemiology has been much more concerned with showing *how* these variables relate than explaining *why* they relate [27]. The formal theoretical approach and ultimate explanatory perspective of behavioral ecology may bring useful tools to bring to bear.

There are a number of existing theoretical resources to draw upon. Evolutionary biologists have paid close theoretical attention to problems in the evolution of ageing and senescence which have an analogous structure to the present one [28], [29], [30]. The models they have employed rely on a distinction between extrinsic and intrinsic mortality. Extrinsic mortality is mortality from sources which cannot be mitigated by anything the organism does, whereas intrinsic mortality is mortality that can be reduced by allocating energy to doing so, for example by repairing tissues or performing avoidance behavior. The general finding of the models is that the prevailing rate of extrinsic mortality sets a limit on how much energy it is worth allocating to mitigating intrinsic mortality. This is intuitive; one would not spend too much on repairing a car in an environment where cars are frequently stolen anyway. The interplay between extrinsic mortality and energy allocated to self-repair leads to the kind of exacerbatory dynamic relevant here, namely that, where extrinsic mortality is high, organisms are selected to invest relatively little in self-repair, and consequently, they senesce early, even if they survive all of the extrinsic hazards of their environment. It may be that behavioral plasticity and social learning are doing something rather similar concerning health behavior in socioeconomically deprived communities as natural selection of genes does in species facing high-mortality regimes, namely finding an adaptive equilibrium with relatively low devotion of energy to self-care.

Mathematical epidemiologists and health economists have also considered optimal (i.e. utility-maximising) choices in the domain of health behavior, particularly in the context of infection risk for sexually transmitted diseases [31], [32]. These models show that the incentive for risk -reduction behavior is much lower for individuals who have a high probability of already being infected, or whose overall mortality rate from all causes is higher, than individuals who are at lower existing risk. This can also create an exacerbatory dynamic, where people already likely to be infected have no incentive to reduce their subsequent infection risk. Thus, the theoretical tools needed for the current question already exist, but they have not been unified and applied to the specific issue of socioeconomic gradients in health behavior before.

In view of the foregoing discussion, a simple theoretical account of the relatively reduced health behavior of people of lower socioeconomic position can be constructed, using the following assumptions:

1. There are primary, unavoidable health effects of low socioeconomic position, because lower socioeconomic position is associated with exposure to more environmental harms over the life course;
2. Because of these primary effects, the payoff for preventative health behavior is reduced, and therefore the optimal amount of preventative health behavior to perform is reduced. This creates a secondary effect, where people of lower socioeconomic position invest less in health behavior;
3. Because the primary and secondary effects are additive, there is an overall socioeconomic discrepancy in final health outcomes which is greater than the primary discrepancy in environmental conditions. This is the exacerbatory dynamic of poverty described at the beginning of the paper.

This verbal account seems intuitively appealing, and is consistent with ethnographic descriptions of attitudes to life in communities facing extreme poverty or danger [33], [34]. However, the claim that not investing in health behavior could actually be optimal in any sense is a strong one, and thus, a formal model is needed to test whether (or rather, under what assumptions) the account sketched above is in fact plausible. In particular, the theory seems potentially convincing for extreme cases, where people are facing such dire circumstances that they are unlikely to survive from year to year anyway, but it is less clear it could account for the persistence of marked socioeconomic differentials in health behavior in societies which are affluent overall and whose material conditions, even for the poorest citizens, are benign by historical standards.

## Methods

The mathematics of the model are presented in Appendix S1. Here, I outline its main elements verbally. I model an individual who faces a certain chance of dying each year. The total mortality risk can be decomposed into two components. The extrinsic mortality rate, *m*, is the probability of being killed by some factor whose probability is not affected by health behavior. The intrinsic mortality rate, *i*, is the probability of dying from some cause for which performing health behavior makes some difference. I conceptualise health behavior as a unitary continuous variable, such that an individual can perform either more or less of it through her life, with the overall amount performed, *h,* expressed in arbitrary units. I assume that the relationship between the amount of health behavior performed and the rate of intrinsic mortality shown is a negative exponential: that is, performing no health behavior means certain death in the first year, and performing ample health behavior reduces intrinsic mortality risk to near 0. The main conclusions of this paper do not rely on the function being this exact shape, as long as more health behavior is associated with less intrinsic mortality risk, but this shape produces particularly neat conclusions.

I also assume that time and energy devoted to health behavior cannot be devoted to other activities which are important to the individual's overall fitness, such as gaining status, allies and resources, finding a mate, looking after family members, and so on. Overall fitness is the product of years alive and the amount of these other activities the individual has managed to perform, so there is a trade-off between performing health behavior and pursuing other components of fitness. I capture the strength of this trade-off with a parameter *α*. The question is: what is the level of health behavior which maximises the individual's fitness, and how does this optimum vary as the rate of extrinsic mortality changes?

## Results

**Figure 1:**
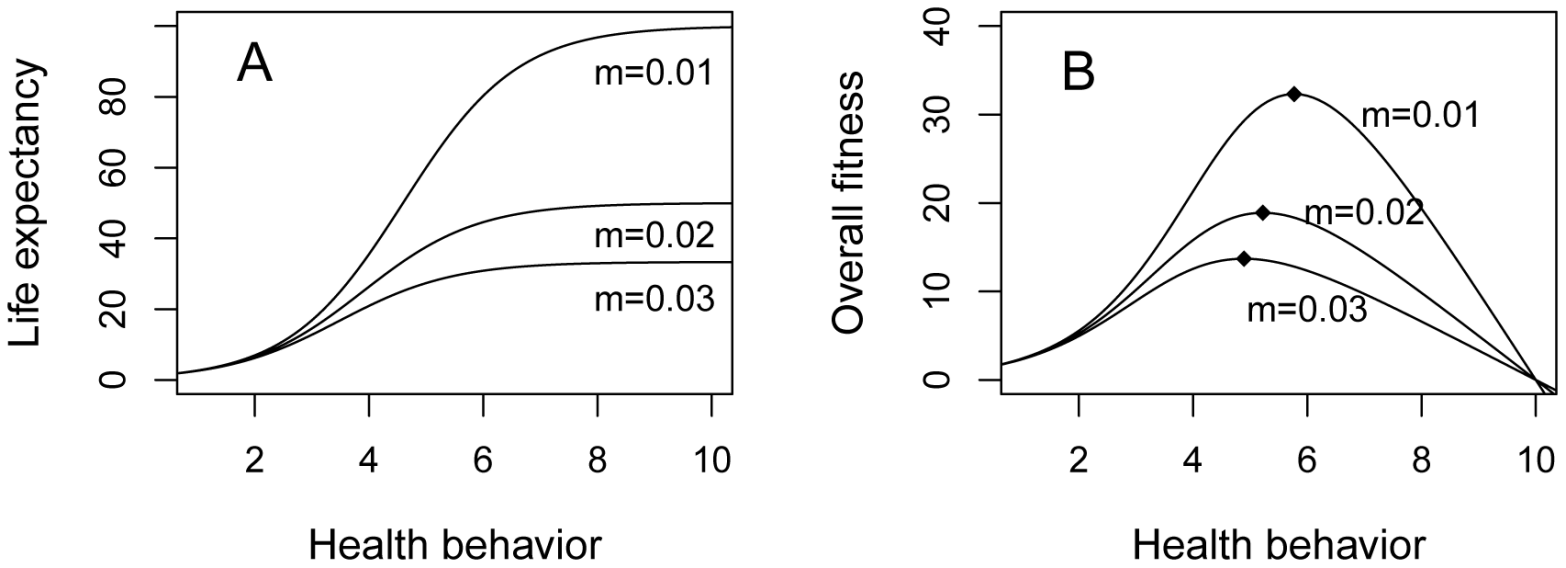
(a) The effect of different amounts of health behavior on life expectancy for three different values of the extrinsic mortality rate *m.* (b) The relationship of overall fitness to the amount of health behavior performed, for three different values of the extrinsic mortality rate *m*, with α = 0.1. Note that as *m* increases, the maximum fitness (shown by the small diamonds) is not only less, but occurs at a lower level of health behaviour.

**Figure 2:**
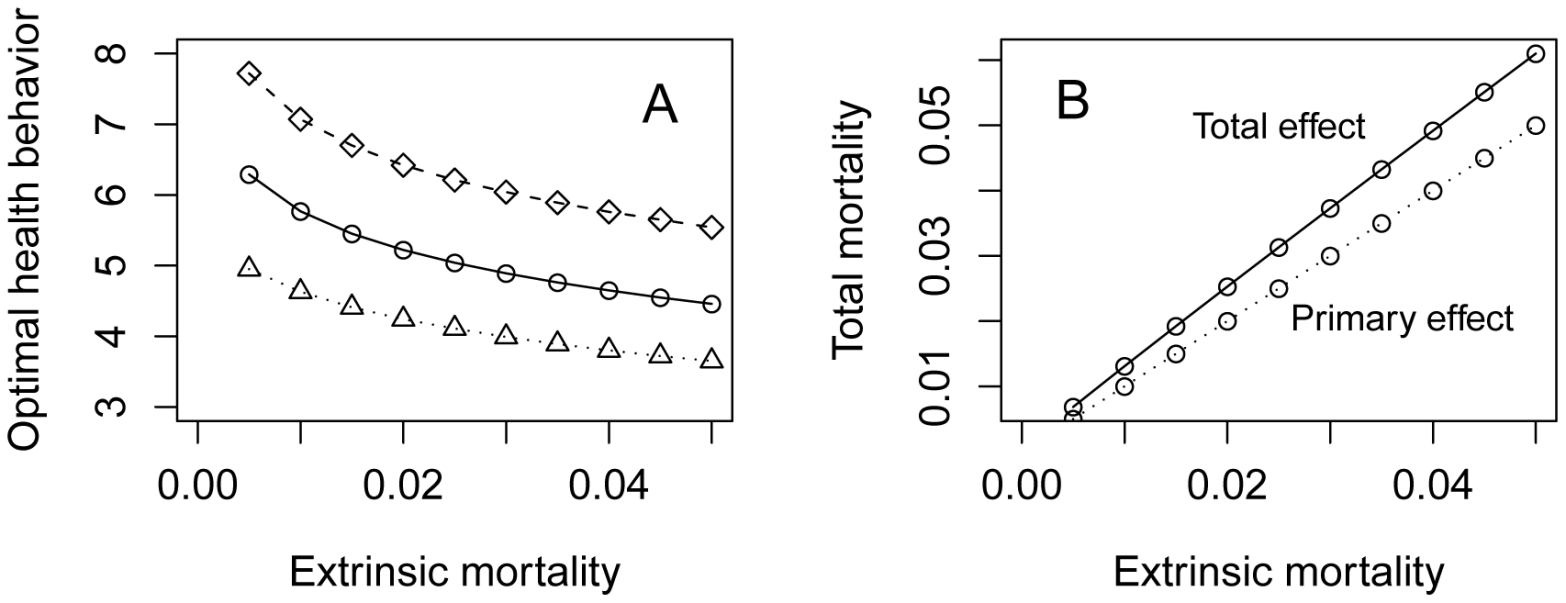
(a) The relationship between the extrinsic mortality rate and h*, the fitness-maximizing amount of health behavior, for three different values of the parameter α, which sets the strength of the trade-off between health behavior and other components of fitness. Diamonds: α = 0.05; Circles: α = 0.1; Triangles: α = 0.15 (b) The predicted effect of increasing the extrinsic mortality rate on total mortality (with α = 0.1). Extrinsic mortality has a primary effect (lower line), but also a secondary effect via reducing the optimal amount of health behavior. The sum of the primary and secondary effects is shown by the upper line.

First, I examine the effect of the amount of health behavior a person performs on their life expectancy, under three different extrinsic mortality rates (figure 1a). Note that *m = 0.*01 means unavoidable mortality events befalling someone every one hundred years on average, *m = 0.02* every fifty years, and *m = 0.*03 every 33.3 years. From the figure, we can see that more health behavior does mean longer expected life, but only to a limit set by *1/m*. Thus, the difference in life expectancy achieved by performing ample health behavior versus none at all is strongly and inversely dependent on the extrinsic mortality rate. Figure 1b shows overall fitness against the amount of health behavior the individual performs, for a representative value of α. The relationship between health behavior and fitness is inverse U-shaped: at first, increasing health behavior steeply increases fitness through increased life expectancy, but as the increases in life expectancy flatten off, subsequent increases in allocation to health behavior actually reduce fitness through their negative impact on other components. There is a clear optimum amount of health behaviour, henceforth designated h*, which is at an intermediate level. The value of h* decreases with increasing extrinsic mortality. This can be seen on figure 1b by the fact that the peak fitness for *m = 0.03* is not only lower than that for *m = 0.02*, but also shifted to the left. Figure 2a illustrates the effect of increasing the extrinsic mortality rate on h* across a wider range of values of *m*, and for three different values of *α*. Increasing *α* strengthens the trade-off between health behavior and other components of fitness, and thus reduces the optimal amount of health behavior h*, but for any given *α*, the negative relationship between *m* and h* is found. The decline in h* is particularly steep as *m* increases from near zero.

If people perform the optimal amount of health behavior given by h* for the particular extrinsic mortality regime that they are experiencing, what will be the consequence? When the rate of extrinsic mortality increases, there will be a primary effect of this on total mortality rates and life expectancies, but also a secondary effect, whereby h* is reduced, and so people perform less health behavior, and so total mortality rates go up further. Thus, as the rate of extrinsic mortality increases, the rate of total mortality is predicted to increase at a faster rate, because of the combination of the primary effect and the exacerbatory secondary effect (figure 2b). This amounts to the exacerbatory dynamic discussed in the introduction: where extrinsic vulnerability is high, we predict a lack of self-care which makes its effects even worse than they otherwise would be.

## Discussion

This very simple model provides a clear ultimate account of why we might expect people living under conditions of social disadvantage to take less care of their health than their more affluent peers. If it is the case that lower socioeconomic position is associated with a greater rate of extrinsic hazards (an assumption which needs justifying, see below), then we should expect people to respond to lower socioeconomic position with reduced preventative health behavior, because the benefits of that behavior to them are indeed lessened. This would in turn make their health outcomes worse, and so the gradient in health outcomes should in general be steeper than the underlying gradient in extrinsic risk exposures. Thus, the observed pattern of substantial socioeconomic gradients in health, which are to a significant extent mediated by differences in health behavior, is exactly what we would predict if people are behaving adaptively given the environment in which they live.

Note that the predicted relationship between health behavior and environmental conditions is a smooth one, not a step or threshold function. This is important because social gradients in health behavior and outcomes are continuous and finely graded [35]. Also, the absolute rates of extrinsic mortality do not have to be high to affect expected health behavior. Thus, this is not just a model of what might be expected to occur under extreme conditions of danger and deprivation. Indeed, given the shape of the relationship between extrinsic mortality and optimal health behavior, it is when extrinsic mortality is low that small differences in it have the greatest effect on predicted health behavior. For example, inspecting figure 1b, increasing the extrinsic mortality rate from 0.5% to 1% has a much more dramatic impact on the optimal amount of health behavior than increasing it from 4.5% to 5% does. Thus, it should really be in affluent societies where overall extrinsic mortality rates are low, that the consequences of within-society variation should produce the most visible behavioral effects. This may account for the generally perplexing observation that inequalities in outcomes, and particularly in behavior, seem to become more marked as average health conditions improve [4], [5], [6], [19].

### Relationship with previous findings

The model results suggest that the reduced preventative health behavior of people facing socioeconomic adversity could be a comprehensible response to the life situations they face, rather than simply being error or incompetence. Essentially this argument was made in a qualitative way by Lawlor et al. [26] for the particular case of smoking, but the current paper generalises the argument to all health behavior, and more importantly provides a formal model to show that the argument does indeed work. The model predictions are parallel to results seen in models of the genetic evolution of mechanisms of cellular repair and ageing [29], [30], and of rational choice of infectious disease risk [31], [32]. Thus, the novelty here is in the application of the framework to the domain of decisions about preventative health behavior, rather than the development of the framework itself.

Very similar behavioral-ecological reasoning to that presented here can also be used to understand social variation in other aspects of behavior, such as age at first childbearing [36], [37], [38], duration of breast-feeding and parental investment in general [39], and risk-taking behaviors [38]. Thus, it is possible that extrinsic mortality may emerge as a key variable for understanding why a whole suite of behaviors – early childbearing, short breastfeeding, reduced preventative health behavior, and risk-taking – are associated with low socioeconomic status in affluent populations [40].

Previous research on social inequalities in health behavior has found that people faced with socioeconomic deprivation endorse a greater belief in the influence of chance on life outcomes, particularly in the domain of health [18], are more pessimistic [21], and devalue future outcomes relative to present ones more sharply [22], [23], [24], than people of higher socioeconomic position. The model presented here is not in any sense an alternative to these accounts. On the contrary, the model here suggests an ultimate reason why these proximal psychological patterns might persist, and the proximal psychological accounts suggest how the adaptive behavior might actually be delivered. Clearly, people do not perform exact actuarial calculations in deciding whether to adopt a particular health behavior. Instead, they presumably employ some simple evolved heuristics [41]. In this case, these might include something like ‘to the extent you see bad and unpredictable health outcomes besetting your peers, worry about today rather than tomorrow’. This would deliver roughly the behavior that the model predicts as optimal.

### Are the assumptions reasonable?

An *a priori* model is only as useful to the extent to which its assumptions capture important features of the situation studied. Of the various assumptions made here, two are arguable and critical to the result: the assumption that lower socioeconomic position means a greater rate of extrinsic hazard, and the assumption that health behaviors have some cost in terms of other components of fitness.

Several lines of evidence suggest that the assumption that lower socioeconomic position is associated with a greater degree of extrinsic hazard may not be unreasonable. First, studies of health inequalities generally find that controlling for behavioral factors (smoking, diet, etc.) attenuates socioeconomic gradients in health outcomes, but does not abolish them entirely [15], [42]. Of course, this could simply mean that not enough controls have been included, but it could also suggest that there is a residuum of health hazard which is extrinsic and thus not responsive to individuals' behavioral decisions. Second, there are some health risk factors whose spatial distribution is socioeconomically patterned, and which people living in more deprived areas can do very little to avoid save for not living there. The clearest examples are noise, lead, and air pollution in the form of fine particles and nitrogen oxides. The levels of these hazards are higher in poor neighbourhoods [43], [44], and their effects on morbidity and mortality well established [43], [45], [46], [47]. Third, many studies have found effects of living in poor neighbourhoods on health outcomes, above and beyond the effects of individual-level socioeconomic characteristics [48]. For example, poorer neighbourhoods are associated with substantially increased chances of accidental death or homicide [49], and heart disease [50], even once individual characteristics are adjusted for. This suggests that there are hazards fundamentally associated with living in these areas, which affect whoever it is that lives there. Finally, adult decisions about health behavior are made in the context of the person's prior developmental history. Early-life factors such as low intrauterine growth restriction, lack of breastfeeding, poor diet in infancy, and so on are effectively extrinsic as far as an adult is concerned, since they have already happened long in the past, but they will powerfully influence future health prospects [51], [52]. Individuals from poor backgrounds are differentially likely to have already been exposed to such hazards [53], [54].

The second key assumption of the model is that performing health behavior has some cost in terms of other components of fitness. If this assumption is relaxed, then the model would predict that individuals should always perform the maximum possible amount of health behavior, regardless of the rate of extrinsic mortality. Although I stressed that the health behaviors of interest here are not those which cost money, there are costs of other kinds. For example, drinking and smoking can service social relationships, risk-taking can enhance social reputation, and so on, and these other activities are clearly very important to a person's reproductive success. Time and energy devoted to a particular health behavior cannot be allocated elsewhere. Thus, it seems reasonable to argue that performing the maximum possible amount of health behavior carries some kind of fitness cost.

### Limitations of the modelling approach

The model presented here is a highly simplified optimization model of the kind which has often been used in life-history theory [55], [56]. It assumes that the rate of extrinsic mortality is set for the individual's life, that it is age-independent, and that the individual has to adopt a single rate of health behaviour for life. Clearly, these assumptions are not realistic, and it would be of interest to use state-dependent models [57] to examine how, for example, decisions about health behavior in the next year should be predicted to vary with health status in the current year. Cichon [30] has presented such an approach for the evolution of allocation of energy to self-repair. He does indeed find that self-repair is predicted to vary with age and state. However, he also finds a general inverse relationship between optimal amount of self-repair and the rate of extrinsic mortality. This accords with the main result discussed here, and suggests that the model presented here is adequate to capture the key qualitative pattern, the socioeconomic gradient, which was my starting point.

### What are the implications?

Whilst the model is satisfying in that it predicts the exacerbatory dynamic of poverty which we observe empirically, a stronger test of its utility is whether it has any practical implications. The model accounts quite naturally for the observation that providing health information or voluntary screening services can actually increase social inequalities in health, since they are taken up differentially by those of higher socioeconomic position [20]. This is because the expected benefit of adopting new health-promoting behaviors, other things being equal, will be greatest for those individuals experiencing the lowest extrinsic mortality rate. Thus, interventions based on legislation or financial incentive may be relatively more effective in deprived social groups than those based on voluntary uptake.

In general, the model presented here draws the focus of health policy away from merely providing information or exhorting behavioral change, and onto extrinsic mortality. As with other neo-material approaches to health inequalities [58], it reminds us of the need to address the fundamental economic inequities which mean that some neighbourhoods contain higher risks of pollution, toxicity, and accident than others. More specifically, it suggests that reducing these structural inequities will reap a double dividend. It will have a primary effect on mortality inequality, and also a secondary effect as people respond to the primary effect by increasing their health-promoting behavior. Indeed, the secular trend in health behavior amongst middle-class people could be interpreted in this way. As economic development has eliminated many of the uncontrollable sources of danger, individuals have increased their investment in behaviors that mitigate those risks which do respond to individual choice. We need to create a similar dynamic in the most disadvantaged areas.

However, whilst changing structural conditions is the most important priority, the model also suggests that it is worth paying attention to people's *perceptions* of extrinsic mortality. That is, in poor communities, individuals may perceive the local environment to be extrinsically dangerous to a greater extent than is in fact true (for example, because they are affected by social stereotypes or media portrayals). The model suggests that the psychological mechanisms which underlie behavioral decisions should be responsive to perceived levels of extrinsic mortality. If these perceptions are unrealistic, then they may lead to excessive fatalism and consequent disinvestment in health behavior. Thus, researchers and practitioners could usefully examine the genesis and malleability of people's perceptions of the extrinsic dangers of their environments, and the relationships of these to their health attitudes and health behaviors.

## Supporting Information

Appendix S1 Mathematical model.
(0.44 MB PDF)
