# Supplementary material for: Why Are There Social Gradients in Preventative Health Behavior? A Perspective from Behavioral Ecology
Source: PLoS One. 2010 Oct 13;5(10):e13371. doi: 10.1371/journal.pone.0013371 (PMC2954172; doi:10.1371/journal.pone.0013371)
Supplement: Appendix S1 — Mathematical model. (0.44 MB PDF) [file pone.0013371.s001.pdf]

Appendix S1: Mathematical Model

Assume an individual who faces two kinds of hazards of dying each year: Extrinsic hazards occur with probability *m* and cannot be mitigated by behavior, whereas intrinsic hazards occur with probability *i*, but *i* depends on the amount of preventative health behavior performed, *h*. Specifically:

$$i = e^{-h} \tag{1}$$

The total probability of dying in a year is thus a function of the amount of health behavior performed, and is given by:

$$t(h) = m + (1-m)i \tag{2}$$

which is:

$$t(h) = m + (1-m)e^{-h} \tag{3}$$

Thus, life expectancy is given by:

$$l(h) = \frac{1}{m + (1-m)e^{-h}} \tag{4}$$

The relationship between *h* and *l(h)* is plotted in figure 1a for three different levels of *m*. Now assume that overall fitness is the product of the number of years that an individual remains alive, and the amount of activity other than health behavior (gaining resources, status and allies, mating, parental effort etc.) that the individual performs, on average, per year of life. However, since time and energy are limited, the more effort the individual allocates to health behavior, the less can be allocated to all these other things. Specifically, performing a level of health behavior *h* means that the amount of other fitness-directed behaviors which can be undertaken is $(1-\alpha h)$, where α is a scaling parameter representing the degree to which performing health behavior impacts negatively on other components of fitness. Thus, overall fitness is a function of health behaviour, given by the expectation of life

(equation 4) multiplied by *(1-αh)*. That is:

$$w(h) = \frac{(1-\alpha h)}{m+(1-m)e^{-h}} \quad (5)$$

Figure 1b plots *w(h)* against *h* for three values of *m* (with α=0.1), showing that fitness is maximised at an intermediate level of health behavior which varies with *m*. To find this optimum allocation to health behavior, *h**, we differentiate fitness with respect to *h*. Thus, at *h**:

$$w'(h) = \frac{(1-\alpha h)(1-m)e^{-h}}{(m+(1-m)e^{-h})^2} - \frac{\alpha}{m+(1-m)e^{-h}} = 0 \quad (6)$$

That is, at *h**:

$$(1-\alpha h)(1-m)e^{-h} = \alpha(m+(1-m)e^{-h}) \quad (7)$$

The value of *h** was solved numerically for values of *m* between 0.005 and 0.05 (with α=0.05, 0.1, or 0.15), and these are plotted in figure 2a. Assuming that individuals choose the optimal amount of health behavior *h**, then the total mortality associated with a particular rate of extrinsic mortality *m* would be expected to be:

$$t^* = m+(1-m)e^{-h^*} \quad (8)$$

This quantity is plotted in figure 2b for values of *m* between 0.005 and 0.05, α=0.1.
